# Updated advances of linking psychosocial factors and sex hormones with systemic lupus erythematosus susceptibility and development

**DOI:** 10.7717/peerj.7179

**Published:** 2019-06-25

**Authors:** Qingjun Pan, Xiaoqun Chen, Shuzhen Liao, Xiaocui Chen, Chunfei Zhao, Yong-zhi Xu, Hua-feng Liu

**Affiliations:** Key Laboratory of Prevention and Management of Chronic Kidney Disease of Zhanjiang City, Institute of Nephrology, Division of Nephrology, Affiliated Hospital of Guangdong Medical University, Zhanjiang, China

**Keywords:** Systemic lupus erythematosus, Psychosocial factors, Sex hormones, Risk factors, Therapy targets

## Abstract

Systemic lupus erythematosus (SLE) is a systemic autoimmune disease that primarily affects women, especially those of reproductive age. Genetics, environment, and gene-environment interactions play key roles in the development of SLE. Despite the numerous susceptibility genes of SLE identified to date, gene therapy is far from a clinical reality. Thus, more attention should be paid to the risk factors and underlying mechanisms of SLE. Currently, it is reported that psychosocial factors and sex hormones play vital roles in patients with SLE, which still need further investigated. The purpose of this review is to update the roles and mechanisms of psychosocial factors and sex hormones in the susceptibility and development of SLE. Based on review articles and reports in reputable peer-reviewed journals and government websites, this paper summarized psychosocial factors (e.g., alexithymia, depression, anxiety, negative emotions, and perceived stress) and sex hormones (e.g., estrogens, progesterone, androgens, and prolactin) involved in SLE. We further explore the mechanisms linking these factors with SLE susceptibility and development, which can guide the establishment of practical measures to benefit SLE patients and offer new ideas for therapeutic strategies.

## Introduction

Systemic lupus erythematosus (SLE) is a systemic autoimmune disease with high heterogeneity that predominantly affects women, especially those of reproductive age (Mok, 2018). Several factors are linked to the pathogenesis of SLE, including genetic and environmental factors, along with gene-environment interactions (Mok, 2017). Although many SLE susceptibility genes have been identified through association studies, gene therapy is a long way from clinical application (Mok, 2017). The conventional therapy for SLE is based on a high dose of immunosuppressive drugs; however, the significant side effects associated with this treatment are causing increasing concern, calling for the urgent need to discover new treatment targets. Toward this end, it is essential to comprehensively consider the diversity of risk factors of SLE, and to investigate the underlying mechanisms contributing to these risks.

Currently, numerous studies report that psychosocial factors and sex hormones are important to SLE. However, the interactions between psychosocial factors and sex hormones and SLE are complicated, which still need further investigated. Therefore, this review is aimed to investigate the roles and effects of psychosocial factors and sex hormones in SLE. To facilitate this field of study, we here provide a systematic review of the effects of psychosocial factors focusing on alexithymia, depression, anxiety, negative emotions, and perceived stress, and sex hormones, focusing on estrogens, progesterone, androgens, and prolactin (PRL), in SLE, and explore the mechanisms contributing to these associations.

### Survey methodology

This paper was based on review articles and reports in reputable peer-reviewed journals and government websites. The research was conducted using Medline on OvidSP, PubMed, Google Scholar, website, books, e-books, and reports. The words “systemic lupus erythematosus”, “psychosocial factors”, “sex hormones”, “alexithymia”, “depression”, “anxiety”, “negative emotions”, “perceived stress”, “estrogens”, “progesterone”, “androgens”, “prolactin” and a combination of those were used to retrieve literature from the databases.

### Psychosocial factors in SLE

Disease activity can be triggered in patients with inactive SLE following experiences leading to negative emotions such as severe mental stimulation and excessive emotional repression. Moreover, substantial research on psychological factors of disease conducted in the last two decades has demonstrated a high frequency of alexithymia, characterized by an inability to recognize or describe personal emotions, among patients with chronic autoimmune diseases such SLE and rheumatoid arthritis (RA); this link was further associated with psychological distress and quality of life (QoL) impairment, thereby increasing the risk of medical or psychiatric diseases (Barbosa et al., 2009, 2011; Vadacca et al., 2008, 2014). In SLE, the link with alexithymia was found to be further related to adult attachment disorders (Barbasio & Granieri, 2013). The inability to develop negative emotions through emotional regulation contributes to direct somatic disturbances, which may enhance the physical symptoms in patients with SLE.

In recent years, depression and anxiety have also been suggested to play an important role in the activation of SLE, owing to their associations with proteinuria (Bai et al., 2016). In addition, other forms of depression have been suggested to occur in SLE patients, including minimal or mild depressive symptoms (Bai et al., 2016; Zhang et al., 2017). The form of the symptoms has been correlated with a complicated mix of biological, social, and psychological factors in SLE patients, with more severe depressive symptoms leading to a high average SLE disease activity index score (Alsowaida et al., 2018).

One hypothesis that has been put forward to explain these associations involves the influence of immune-neuro-endocrine factors in the pathogenesis of SLE and RA (Vadacca et al., 2008). Negative emotions have been suggested to influence the immune system via impacting the sympathetic nervous system and endocrine functions, leading to immune dysregulation (Barak, 2006; Dowlati et al., 2010; Matsunaga et al., 2008; Schiepers, Wichers & Maes, 2005; Solomon, Amkraut & Kasper, 1974). Indeed, neuropeptides and endocrine hormones play a crucial role in the pathogenesis of SLE. A multitude of studies revealed that an increased level of neuropeptides decreases the hypothalamic-pituitary-adrenal axis tone, giving rise to abnormalities of immune function and inflammatory processes, thereby contributing to the development of SLE both in patients and animal models (Bracci-Laudiero et al., 1998, 1999; Harle et al., 2006). Depression and perceived stress, as negative emotions, are mediated by feelings of helplessness originating from this altered immune function (Mills et al., 2018; Roberts et al., 2018). Moreover, some studies found that SLE patients frequently produced serum antibodies against N-methyl-D-aspartic acid receptors (Diamond et al., 2006), leading to structural and functional damage of the amygdala in patients and animal models of SLE, which in turn contribute to an associated deficit in emotional processing such as cognitive and emotional abnormalities (Diamond et al., 2006; Watson et al., 2012).

**Figure 1:**
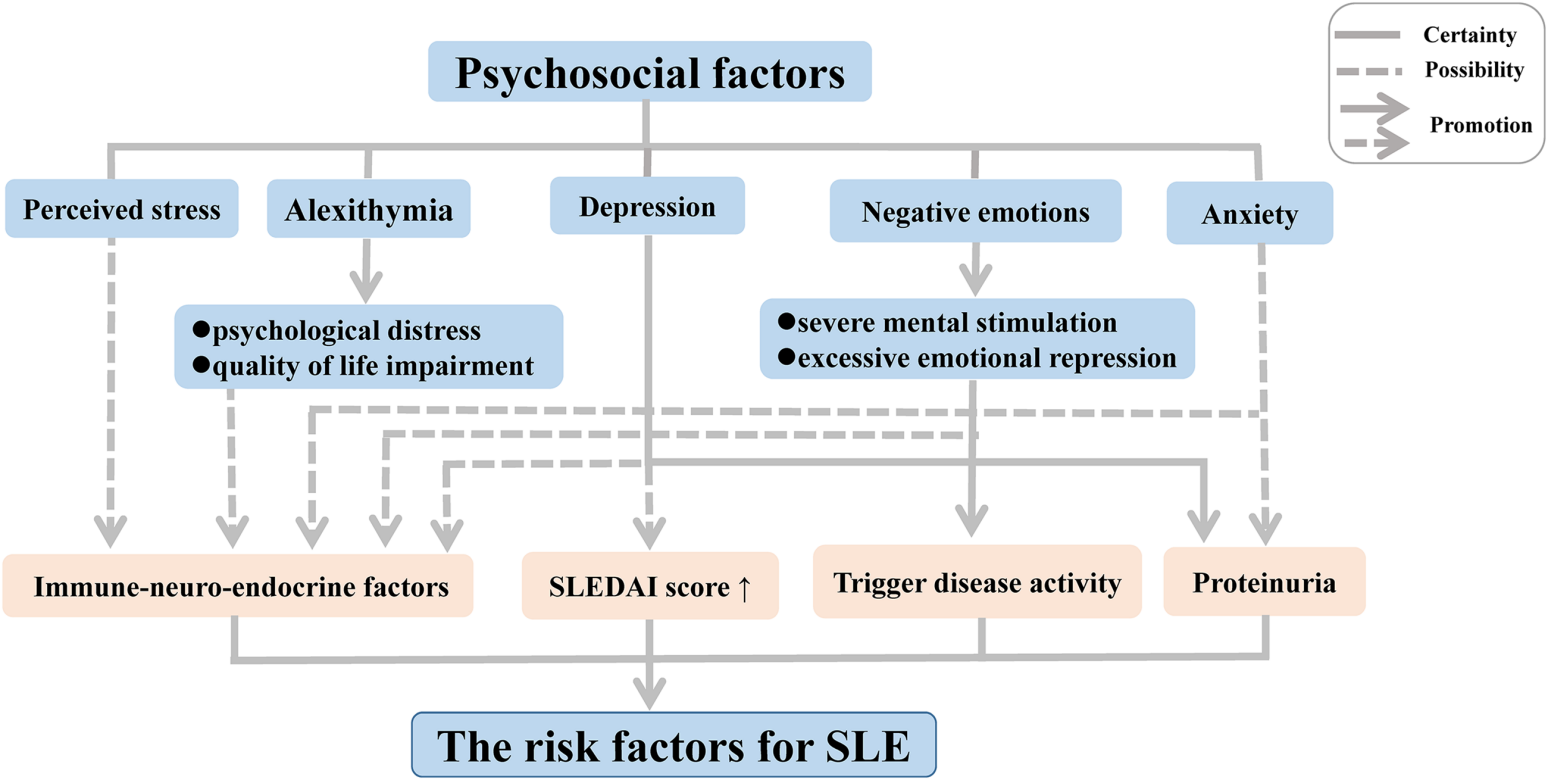
Psychosocial factors as risk factors for SLE. Abbreviation: SLEDAI score, SLE Disease Activity Index score.

Although it has now become clear that psychosocial factors may influence the disease onset and progression of SLE independently and interactively (Fig. 1), the precise biological mechanisms that mediate these responses implicated in the development of SLE remain poorly understood. Thus, further research should focus on elucidation of the immune mechanisms related to alterations in mood and emotion in SLE, which will be indispensable to gain a comprehensive understanding of the complex pathogenesis.

Additionally, finding an efficient way to regulate the psychosocial factors may be good for the physical treatment of the patients with SLE. Mindfulness-based cognitive therapy (MBCT) is a treatment combining mindfulness meditation and interventions from cognitive therapy and is delivered in a group setting (Barnhofer et al., 2009). Recently, a clinical trial found that MBCT contributed to decreased psychological symptoms, such as depression, anxiety and alexithymia, and improved QoL, as well as benefited physical treatments in patients with SLE (Solati et al., 2017). Therefore, MBTC may be a promising intervention for treating psychosocial problems in clinical populations, including patients with SLE, although its efficacy is little known now. At the same time, there will be an increasing intervention to remove psychosocial symptoms for the patients with SLE.

### Sex hormones in SLE

Most human autoimmune diseases show an increased incidence and prevalence in females; this is particularly true for SLE, which is exacerbated during puberty, pregnancy, and post-partum periods (Tedeschi, Bermas & Costenbader, 2013). Many sex hormones that are also known to affect the immune system (Parks et al., 2017; Pennell, Galligan & Fish, 2012), may play a role as triggers or protectors of the development of SLE (Sakiani, Olsen & Kovacs, 2013; Tedeschi, Bermas & Costenbader, 2013). Studies in human populations and experimental models have indicated an elevated risk of SLE associated with exposure to estrogen (Cunningham et al., 2014; Cutolo, Sulli & Straub, 2012; Hughes & Choubey, 2014; Sakiani, Olsen & Kovacs, 2013; Strickland et al., 2012), whereas progesterone and testosterone appear to play a protective role via counteracting the effects of estrogen (Hughes, 2012; Hughes & Choubey, 2014; Sakiani, Olsen & Kovacs, 2013; Tan, Peeva & Zandman-Goddard, 2015; Tsur et al., 2015). Although the risk of developing SLE from exposure to other sex hormones such as PRL remains controversial, a pathogenic role of PRL in SLE can be inferred based on epidemiological and experimental animal studies (Costanza et al., 2015; Karimifar et al., 2013; Orbach et al., 2012; Saha et al., 2011; Shelly, Boaz & Orbach, 2012). In addition, limited data suggest a role of luteinizing hormone releasing hormone, also known as gonadotropin releasing hormone, in the pathogenic mechanisms of SLE (Jacobson, 2000, 2001; Jacobson et al., 1999; Jacobson, Nisula & Steinberg, 1994; Walker & Jacobson, 2000). The influence of these sex hormones on SLE and their effects are discussed in detail below.

#### Estrogens

The potential role of estrogen in autoimmune diseases has been extensively investigated, particularly for SLE. The mode of action of estrogen and its metabolites differs with regards to the specific effects on the modulation of immunity (Cutolo, Sulli & Straub, 2012). It is well established that low doses of estrogen enhance T helper (Th)1 cell responses, whereas high estrogen levels raise Th2 responses (Beagley & Gockel, 2003; Salem, 2004). By contrast to estradiol (E2), estriol (E3) strongly provokes antibody production in response to bacteria (Ding & Zhu, 2008). In genetically predisposed individuals, estrogens increase the risk of disease and have a pathogenic role in SLE. The pathogenic mechanisms may involve the production of type 1 interferon (IFN), survival of auto-reactive B cells and production of pathogenic immunoglobulin (IgG) auto-antibodies, and/or differentiation of CD4^+^ Th cells (Sakiani, Olsen & Kovacs, 2013; Tabor & Gould, 2017). Estrogen signals act on the nuclear receptors estrogen receptor (ER)-α and ER-β in many types of immune cells (Cunningham & Gilkeson, 2011; Hill et al., 2011; Kovats, 2012; Moulton, 2018; Yakimchuk, Jondal & Okret, 2013). Enhanced expression of type 1 IFN-inducible genes has been detected in SLE, and this so-called “IFN signature” has been shown to play a critical role in the pathogenesis of SLE and is associated with active disease (Baccala et al., 2007; Baechler et al., 2003; Li et al., 2010; Ronnblom & Eloranta, 2013). Thus, active immunization with IFN-kinoid can downregulate this IFN signature in SLE (Lauwerys et al., 2013). In a mouse model, estrogens were suggested to increase the production level of IFN-α through up-regulation of genes regulating the production of type 1 IFN such as *Ifi202* (Choubey, 2012; Choubey et al., 2011; Panchanathan et al., 2009), *Unc93b1* (Panchanathan, Liu & Choubey, 2013), or *Irf5* (Choubey et al., 2011; Shen et al., 2010). Simultaneously, type 1 IFNs also increase estrogen signaling in immune cells by provoking the expression of ER-α, thereby contributing to SLE development and progression (Choubey et al., 2011; Panchanathan et al., 2010).

Moreover, numerous studies have suggested that estrogens have various effects on B cell development, survival, and differentiation, and in regulating the production of pathogenic auto-antibodies (Hill et al., 2011). Estrogen promotes the survival of self-reactive B cells by preventing their elimination or inactivation at developmental checkpoints. This is possibly related to the up-regulated expression of Bcl-2, CD22, SRC homology region 2 domain-containing phosphatase-1 (SHP-1), vascular cell adhesion molecule 1 (VCAM-1), protein tyrosine phosphatase, non-receptor type 6 (PTPN6), and B-cell activating factor (BAFF) (Bassi et al., 2015; Bynoe, Grimaldi & Diamond, 2000; Grimaldi et al., 2002; Hill et al., 2011; Panchanathan & Choubey, 2013). However, selective estrogen receptor modulators raloxifene suppressed estrogen-mediated effects on the survival, differentiation, and activation of autoreactive B cells in NZB/WF1 mice, which might serve to ameliorate lupus activity (Zhang et al., 2010).

An Ig class switch occurs in B cells from IgM to IgG, which requires activation-induced cytidine deaminase (AICDA), and estrogens have been shown to increase the expression of AICDA and homeobox protein Hox-C4 (HOXC4) in B cells, leading to the development of pathogenic IgG auto-antibodies (Mai et al., 2010; Pauklin et al., 2009; Sakiani, Olsen & Kovacs, 2013). Estrogens can also enhance the production of anti-double-stranded DNA (dsDNA) antibody and IgG or IgM by peripheral blood mononuclear cells and serum, which enhances the disease severity causing flare-ups (Khan & Ansar Ahmed, 2016). Moreover, estrogen has multiple effects on the development, differentiation, and functions of CD4^+^ T cells (Walters et al., 2009). In SLE, estrogen induces activation of the T lymphocytes via ER-α and ER-β, and increases the expression of T cell activation markers such as CD154 and calcineurin (Lin et al., 2011; Rider et al., 2006). Estrogen was also shown to exacerbate lupus disease severity via an ERα-independent mechanism along with other immune effects contributing to lupus pathogenesis, including modulation of Toll-like receptor (TLR) pathways, dendritic cell development, or E2-TWEAK signaling (Scott et al., 2017). These mechanisms might also contribute to the pathogenic mechanism of SLE, primarily affecting women.

#### Progesterone

The relationship between progestogens and SLE is complex. Progesterone has been suggested to play an important protective role against SLE disease activity (Hughes, 2012; Hughes & Choubey, 2014; Tan, Peeva & Zandman-Goddard, 2015; Tsur et al., 2015). Progesterone can also counteract the effects of estrogens summarized above, such as the estrogen-induced increase in type 1 IFN production, survival of auto-reactive B cells and pathogenic IgG auto-antibodies production, and differentiation of CD4^+^ Th cells. Progesterone has immune-modulatory effects that generally have anti-inflammatory outcomes (Tait, Butts & Sternberg, 2008). In SLE, the promotion of IFN-α/β production is primarily controlled by IFN regulatory factors (IRFs) such as IRF-3, IRF-5, and IRF-7 (Baccala et al., 2007; Fu et al., 2011; Niewold et al., 2012; Ronnblom, Eloranta & Alm, 2006; Ronnblom & Pascual, 2008; Sigurdsson et al., 2005; Stone et al., 2012; Wang et al., 2013). Progestogens impair IRF-7 activation in plasmacytoid dendritic cells (Hughes et al., 2008), which regulate the TLR-mediated decreased production of IFN-α, a major source of type 1 IFN, through depot medroxyprogesterone acetate (DMPA), thereby ameliorating the IFN signature and consequently SLE disease activity (Tan, Peeva & Zandman-Goddard, 2015). Moreover, progesterone has the opposite effect of estrogen with regards to the Ig class switch of B cells, resulting in a decrease of the IgM to IgG class switch recombination via suppressing the transcription of AICDA (Park, 2012; Pauklin & Petersen-Mahrt, 2009; Pauklin et al., 2009; Sakiani, Olsen & Kovacs, 2013). Specifically, progesterone reduces the production of IFN-γ, which induces a class switch to IgG2a in B cells, leading to pathogenic IgG auto-antibodies production (Hughes, Clark & Wong, 2013). In the last decade, there has been much research focused on the promotion effect of progesterone for Th2 differentiation; in particular, high levels of progesterone will induce a Th2-type immunologic response and suppress a Th1-type response via inhibiting IL-12 signaling in SLE (Hughes, Clark & Wong, 2013; Miyaura & Iwata, 2002). Meanwhile, there is a decrease in mortality, glomerulonephritis and Th1-related autoantibody production after DMPA treatment in NZB/NZW mice, which suggest that progesterone may have therapeutic benefit for SLE patients (Hughes et al., 2009; Recalde et al., 2018).

#### Androgens

Androgens may play an important protective role in the pathogenic mechanisms contributing to the development of SLE. Similar to progesterone, androgens target key immune pathways that can protect against SLE by counteracting the effects of estrogens. In particular, androgens down-regulate the levels of *Ifi202*, which in turn decreases the production of type 1 IFN (Choubey, 2012; Choubey et al., 2011; Panchanathan et al., 2009). Compared with estrogens, androgens may enhance the checkpoints for the auto-reactivity of B cells to induce B cell apoptosis via decreasing the level of Bcl-2 in B cells (Altuwaijri et al., 2009), and can also increase the level of transforming growth factor beta 1 (TGF-β1) in marrow stromal cells (Olsen, Gu & Kovacs, 2001), thereby suppressing the development of autoimmunity (Gubbels Bupp & Jorgensen, 2018; Sakiani, Olsen & Kovacs, 2013; Zhu et al., 2016). Androgens have also been shown to decrease the level of pathogenic IgG auto-antibodies production through inhibition of Ig class switching (Sakiani, Olsen & Kovacs, 2013). Androgens decrease the Th1 differentiation of CD4^+^ T cells via inhibiting IL-12 signaling, which in turn mitigates CD4^+^ responses in autoimmune disease (Kissick et al., 2014). Androgens also appear to modulate the accumulation of Gr1^+^CD11b^+^ cells in male mice and could inhibit the function of T follicular helper cells, formation of the germinal center, and the differentiation of plasma cells (Bird et al., 2017). CD11b^+^ cells were shown to overexpress DHT-regulated genes and colony-stimulating factor 3 receptor (Gubbels Bupp, Jorgensen & Kotzin, 2008), which was identified to influence the development of lupus and RA (Elera-Fitzcarrald et al., 2017; Gubbels Bupp, Jorgensen & Kotzin, 2008).

#### Prolactin

Only very limited data are currently available on the contributions of PRL to the development of autoimmune diseases. Indeed, the relationship between PRL and SLE and the underlying mechanisms remain controversial; however, the general perception is that PRL does play a pathogenic role in SLE, particularly with respect to hyper-prolactinemia (Costanza et al., 2015; Karimifar et al., 2013; Shelly, Boaz & Orbach, 2012). Under a specific genetic background, like estrogen, PRL has also been shown to promote the survival of self-reactive B cells by impairing B cell receptor-mediated clonal deletion and decreasing B cell apoptosis, thus breaking down B cell self-tolerance, leading to the development of autoimmunity (Elera-Fitzcarrald et al., 2017; Gonzalez, Saha & Peeva, 2013; Orbach & Shoenfeld, 2007; Saha et al., 2009, 2011). In B6.Sle3 mice, PRL increases the expression of co-stimulatory molecules (CD40, CD86 (B7-2), and MHC II molecules) on B cells, leading to enhanced antibody responses (Gonzalez, Saha & Peeva, 2013; Matera, Mori & Galetto, 2001). With respect to the differentiation of CD4^+^ Th cells, PRL up-regulates the levels of INF-γ, IL-12, IL-2, and CD40L (Clevenger, Altmann & Prystowsky, 1991; Clevenger et al., 1990; Gonzalez, Saha & Peeva, 2013; Jara et al., 2017; Matalka, 2003; Mukherjee, Mastro & Hymer, 1990; Orbach et al., 2012), leading to CD4^+^ T cell activation that can drive the development of autoimmunity in SLE. Recent studies have also implicated PRL in STAT5 activation, and in the induction of Ig synthesis and anti-dsDNA antibodies in SLE. However, it is reported that bromocriptine, a drug that inhibits PRL secretion, abrogates some of the immune effects of PRL in mice (Saha et al., 2011). Actually, the association between PRL levels and disease activity in SLE is still controversial (Elera-Fitzcarrald et al., 2017). Thus, further research on these relationships is warranted.

Here, we updated the roles and the effects of sex hormones including estrogens, progesterone, androgens, PRL on SLE, and some feasible measures could suppress some of effects of sex hormones in SLE mice. Most importantly, the mechanisms linking these factors with SLE susceptibility and development were explored (Table 1), which can guide the establishment of practical measures to benefit SLE patients and offer new ideas for therapeutic strategies.

**Table 1:** Influence of sex hormones on SLE and their effects.

Sex hormones	Regulation	Performance	Effects in SLE
Estrogens	*Ifi202*↑, *Unc93b1*↑, or *Irf5*↑	IFN-α↑, type 1 IFN↑	Pathogenic effect
	Bcl-2↑, CD22↑, SHP-1↑, VCAM-1↑, PTPN6↑, BAFF↑	Survival of auto-reactive B cells↑	
	AICDA↑, HOXC4↑	Pathogenic IgG auto-antibodies↑	
	ER-α↑, ER-β↑, CD154↑, calcineurin↑	Differentiation of CD4^+^ Th cells↑	
Progesterone	IRF-7 activation↑	IFN-α↓, type 1 IFN↓	Protective effects
	IFN-γ↓, suppressing the transcription of AICDA	Pathogenic IgG auto-antibodies↓	
	Inhibit IL-12 signaling	Induce Th2-type immunologic response and suppress Th1-type response	
Androgens	*Ifi202*↓	Type 1 IFN↓	Protective effects
	Bcl-2↓	Survival of auto-reactive B cells↓, TGF-β1↑	
	Inhibit Ig class switching	Pathogenic IgG auto-antibodies↓	
	Inhibit IL-12 signaling	Th1 differentiation of CD4^+^ T cells↓	
Prolactin (PRL)	BCR-mediated clonal deletion, B cell apoptosis↓	Survival of auto-reactive B cells↑	Pathogenic effect
	INF-γ↑, IL-12↑, IL-2↑, CD40L↑	CD4^+^ T cell activation	
	Ig synthesis↑, anti-dsDNA antibodies↑		

**Note:**

Abbreviation: LHRH, luteinizing hormone releasing hormone; GnRH, gonadotropin releasing hormone.

## Conclusions

To sum up, we summarized psychosocial factors (including alexithymia, depression, anxiety, negative emotions, perceived stress) and sex hormones (including estrogens, progesterone, androgens, PRL) affecting the susceptibility and development of SLE. Detailed investigations of the roles and effects of psychosocial factors and sex hormones in SLE could provide guidance for possible prevention or treatment measures. Psychosocial factors may impact the progression and susceptibility of SLE. With the removal of psychosocial symptoms, patients with SLE may get better physical treatments. Sex hormones also influence the development and susceptibility of SLE. Some sex hormones (e.g., estrogens, PRL) may act as risk factors for SLE, and some (e.g., progesterone, androgens) may keep protective effects in the development of SLE. The potential mechanisms of them in SLE, such as provoking antibody production and promote the survival of self-reactive B cells, are complicated. Recent research in this regard has offered insight into the biological mechanisms underlying these associations. Thus, practical methods targeting these risk factors based on gaining a greater understanding of the pathogenic mechanism will benefit SLE patients and may provide new therapeutic strategies.

## Additional Information and Declarations

### Data Availability

The following information was supplied regarding data availability: The research in this article did not generate any data or code; this article is a literature review.
